# 

**Published:** 1894-01-06

**Authors:** 


**Notice to Advertisers and Subscribers.**

All Advertisements, Orders for Copies of THE HOSPITAL, and Business Communications should be addressed to The Publisher, *not to the Editor*, THE HOSPITAL, 428, Strand, W.C.

The Cost of Subscription, post free, is as follows:—Per week, 2½d.; per quarter, 2s. 6d.; per half-year, 5s.; per annum, 8s. 6d. Foreign:—Per Annum, 10s. 8d.; payable, in all cases, in advance.

**OUR NEW OFFICES.**

428, Strand, W.C., will henceforth be the Office of this Journal.

**Notices of Births, Deaths, and Marriages** are inserted in this column at a charge of 2s. 6d. for an announcement not exceeding four lines.

### NOTICE TO CORRESPONDENTS.

All MS., Letters, Books for Review, and other matters intended for the Editor should be addressed THE EDITOR, THE LODGE, PORCHESTER SQUARE, LONDON, W.

The Editor cannot undertake to return rejected MS., even when accompanied by stamped directed envelope.

**"Burdett's Hospital Annual," 1893.**

Fourth year of publication. 700 pp. Boards, gilt lettering. A most complete guide to British, Colonial, Indian, and American Hospitals, Dispensaries, Nursing and Convalescent Institutions, and Asylums. Price 5s. Published by THE SCIENTIFIC PRESS (Limited), 428, Strand, W.C.

**NOTICE.—The Index for Vol. XIV. (ending Sept. 30th, 1893) is on sale at the Office of this Journal, price 2½d., post free.**

### SCRAPS AND GLEANINGS.

The Duke of Saxe-Coburg and Gotha has sent a donation of £50 to the Charing Cross Hospital.

It is intended to hold the annual ball in favour of the funds of the Cardiff Infirmary early this month.

There were treated at the World's Fair Emergency Hospital 18,500 cases, and there were 23 deaths registered at the institution.

Through the will of the late Sir A. Clark the testator bequeaths £500 to the London Hospital Medical College for the foundation of a scholarship.

The autopsy in the case of Prince Alexander of Battenberg showed that the original cause of his illness was the lodging of a cherry-stone in the vermiform appendix.

The Duchess of Teck has sent to the Children's Hospital Home, Kingston Hill, Surrey, a large parcel of warm and useful clothing from the London Needlework Guild.

Towards the funds for the Consumptive Homes of Scotland, a cheque for £1,000 has been received from Liverpool; whilst another anonymous donor in Glasgow has contributed £100 for the same object.

It has been proposed to erect a movable Fever Hospital for the Eastern Ross District (Inverness-shire), which could thus be made available for the four districts, the cost of which would probably be about £200.

After ten years' unsuccessful endeavour the Local Government Board of St. Marychurch, Torquay, have at length finally decided to erect an Infectious Diseases Hospital for that district at a probable cost of £2,200.

The local boards of Acton, Chiswick, and Hanwell have finally selected the hamlet of Perivale, near Ealing, for the purpose of erecting a joint fever hospital. Considerable local opposition to the proposal prevails.

An entertainment was recently given by the employés of Messrs. Stuchbery and Thompson, of High Street, Maidenhead, in favour of the funds of the Cottage Hospital in that town, which benefited to the extent of £12 by the undertaking.

The second series of lectures by the Sunday Lecture Society was inaugurated by Sir Benjamin Ward Richardson, M.D., F.R.S., who lectured on the subject of "The Mastery of Pain," at St. George's Hall, Langham Place, on Sunday, December 10th.

The fever hospital ship "Elk," which we recently announced as having been purchased by the Rochester Port Sanitary authorities for the purpose of isolating infectious cases, has since sunk at her moorings; there were no patients, fortunately, aboard.

The annual meeting of the subscribers to the Dudley Guest Hospital (Birmingham) took place recently. The report of the committee showed that the year's income was £3,161, against £3,454 the previous year, which marked decrease is much to be regretted.

An appeal is now being made in Dublin to enable the authorities of Cork Street Fever Hospital to meet a heavy deficit on the accounts. The admissions for the past year amount to 1,309 patients, which excessive number has caused a great outlay of the institution.

An interesting trial case is being carried on at Portugal on a medical practitioner of that town. Dr. Urbino Prettas has been accused of the intent to poison members of his wife's family, in order to inherit certain money which would have passed to himself had he been left sole survivor.

The Committee of the London Hospital have started a fund in commemoration of the work Sir Andrew Clark has done at the hospital. They intend that the memorial shall take the form of the endowment of a bed or beds. If subscription warrants it, the memorial will take the form of a new ward.

The Princess Edward of Saxe-Weimar opened "The Children's Salon" Sale and Exhibition at St. Martin's Town Hall on Wednesday, 20th inst. An additional cot, endowed by 3,000 little workers in art, literature, and music, will shortly be opened. All articles exhibited and sold on this occasion were the work of the children's own hands.

The report of the Brixham Cottage Hospital states that twenty-six cases had been treated during the past twelve months. The revenue derived from the district nursing work in connection with the establishment forms a substantial supplement to the funds. After payment of all expenses £12 remains to the good on the accounts of this hospital.

The treasurer's report at the annual meeting of the Glasgow Maternity Hospital showed that the ordinary income was £1,696, and the expenditure £2,004. The deficiency of £300 on last year had been supplied by a legacy from the Bellahouston Trustees of £500. The number of women confined under the care of the hospital staff during the year was 2,794.

The foundation of a hospital at Patna, to which we referred as having been due to the exertions of the Church Missionary Society, owed its existence, we learn, entirely to the instrumentality of the Zenana Bible and Medical Mission. This society is doing a good work in India. Last year the number of attendances at the socie y's hospitals and dispensaries numbered 30,730.

Besides the Patna Hospital alluded to, a new one will shortly be erected at Jaffna, where the Zenana Bible and Medical Mission has already established itself. These medical missions are all under the charge of fully-qualified lady doctors, in numbers greatly in excess of any other missionary society.

The After Care Association for Poor Female Convalescents on Leaving Asylums for the Insane. The annual meeting will be held at 83, Lancaster Gate, W., on Monday, January 15th, at three p.m. Sir B. W. Richardson, M.D., will preside. Cards of invitation to be had on application to the Secretary, H Thornhill, Roxby Street, Church House, Dean's Yard, Westminster, S.W. The chief objects of this association are to facilitate the readmission of poor female convalescents from lunatic asylums into social life. There are 30,000 women of various callings in lunatic asylums in England and Wales. The committee have opened a Convalescent Home in Surrey, for which funds are earnestly entreated.

### MEDICAL VACANCIES AND APPOINTMENTS.

### APPOINTMENTS.

E. Baines, B.A., M.B., B.C., appointed House Surgeon to the Sunderland Infirmary. W. P. Blumer, F.R.C.S.Edin., appointed Honorary Surgeon to the Sunderland Infirmary. W. A. Bowring, M.R.C.S., L.R.C.P., appointed Senior Resident Medical Officer to the Royal Free Hospital. R. Campbell, B.A., M.B., M.R.C.S., L.R.C.P., appointed Visiting Surgeon to the Chester General Infirmary. L. V. Cargill, F.R.C.S.Eng., appointed Surgical Registrar to King's College Hospital. W. S. Colman, M.D., M.R.C.P., appointed Assistant Physician to the Hospital for Sick Children, Great Ormond Street. B. Cox, M.B., B.S.Durh., appointed House Physician to the Sunderland Infirmary. H. Gardner, M.R.C.S., L.R.C.P., appointed Resident Medical Officer to the Chelsea Hospital for Women for one year. A. Guennell, M.R.C.S., L.R.C.P., appointed Junior Resident Medical Officer to the Royal Free Hospital. R. Hamilton, M.B., M.R.C.S., L.R.C.P., appointed House Surgeon to the Chester General Infirmary. F. H. Marson, M.B., B.S.Durh., M.R.C.S., L.R.C.P., appointed House Surgeon to the Sheffield General Infirmary. Mr. O. C. Maurice, appointed Assistant Medical Officer of the Infirmary of the Parish of St. Marylebone. H. H. Phillips, M.R.C.S.Eng., L.R.C.P.Lond., appointed House Surgeon to Charing Cross Hospital. A. Routh, M.D., B.S., M.R.C.P., appointed Consulting Obstetrical Physician to the St. Marylebone General Dispensary. W. B. Smith, M.R.C.S., L.R.C.P., appointed District Medical Officer to the Salford Royal Hospital.

### VACANCIES.

The following vacancies are announced this week, the amount of salary in each case being given after the name of the institution:

### Resident Medical Officers.

Central London Ophthalmic Hospital, Gray's Inn Road, W.C. House Surgeon. Applications, with testimonials, to the Secretary before January 8th, 1894.

Horton Infirmary, Banbury. House Surgeon and Dispenser; duly qualified and registered. Salary £60 per annum, with board and lodging. Applications and testimonials to C. H. Davids, 21, Marlborough Road, Banbury, by January 6th, 1894.

Victoria Hospital for Sick Children, Queen's Road, Chelsea, S.W House Surgeon and House Physician to the In-patients. Honorarium £50 each per annum, with board and lodging in the hospital. Applications to the Secretary by January 13th, 1894.

### Non-Resident Medical Officers.

Cosford Union, Suffolk. Medical Officer and Public Vaccinator for the Boxford District. Salary £50 per annum, exclusive of usual extra medical fees. Must reside in the district. Applications to Alfred Newman, Clerk to the Guardians, Union Offices, Hadleigh, Suffolk, by January 11th, 1894.

Dental Hospital of London, Leicester Square. Dental Surgeon; must be a Licentiate in Dental Surgery. Applications to J. Francis Pink, Secretary, by January 8th, 1894.

King's College, London. Curatorship of the Museum. Applications to J. W. Cunningham, Secretary.

Surrey Dispensary, Great Dover Street, S.E. Surgeon. Honorarium £52 10s. per annum. Applications to J. Harrison, 179, Bermondsey Street, S.E., before January 9th, 1894.

Worcester General Infirmary. Physician. Appointment for seven years. Applications to the Executive Committee under cover to the Secretary, Mr. W. Stallard, Worcester Chambers, Pierpoint Street, Worcester, by January 6th, 1894.

### Guthrie Society.—Westminster Hospital.

The monthly meeting of the Society was held as a clinical evening, which, by kind permission of the House Committee, took place in the Board-room at the hospital. The President took the chair at half-past eight. He was supported by the Vice-President and the following members of the staff: Drs. Hebb-Rivers, Pollock, Wills, and Tims; Messrs. Powell and De Santi. Thirty-six other members and visitors were present. The following cases were exhibited: Dr. Marett Tims:—1. Colloid Milium or Hydradenoma (painting); 2. Micro slide of Xanthoma Diabeticorum; 3. Diffuse Symmetrical Scleroderma. Mr. T. W. Bailey:—1. Bronchiectasis with Chronic Pneumonia; 2. Left Hemichorea in Man æt. 25. Mr. W. B. Winckworth:—1. Paralysis Agitans; 2. Disseminated Sclerosis; 3. Lateral Sclerosis; 4. Tabes Dorsalis with Ataxy; 5. General Paralysis of the Insane. Mr. H. C. De Renzi:—1. Amputation of Tubercular Breast; 2. Radical Cure of Hernia (Watson Cheyne's Modification of Bassin). Mr. H. Witham, for Mr. Hartridge, F.R.C.S.:—1. Cololoma of Choroid Viris; 2. Thromlosed Retinal Vein. Mr. Clay:—1. Diaphragmatic Palsy and Supposed Sarcomatous Growth in Right Iliac Region. Mr. Powell, F.R.C.S.:—Congenital Double Dislocation of Gemora.

